# Zinc depletion promotes apoptosis-like death in drug-sensitive and antimony-resistance *Leishmania donovani*

**DOI:** 10.1038/s41598-017-10041-6

**Published:** 2017-09-05

**Authors:** Shalini Saini, Kavita Bharati, Chandrima Shaha, Chinmay K. Mukhopadhyay

**Affiliations:** 1 Special Centre for Molecular Medicine, Jawaharlal Nehru University, New Delhi, 110067 India; 2 National Institute of Immunology, Aruna Asaf Ali Marg, New Delhi, 110067 India

## Abstract

Micronutrients are essential for survival and growth for all the organisms including pathogens. In this manuscript, we report that zinc (Zn) chelator N,N,N’,N’-tetrakis(2-pyridinylmethyl)-1,2-ethylenediamine (TPEN) affects growth and viability of intracellular pathogen *Leishmania donovani* (LD) by a concentration and time dependent manner. Simultaneous addition of zinc salt reverses the effect of TPEN. Further experiments provide evidence of apoptosis-like death of the parasite due to Zn-depletion. TPEN treatment enhances caspase-like activity suggesting increase in apoptosis-like events in LD. Specific inhibitors of cathepsin B and Endoclease G block TPEN-induced leishmanial death. Evidences show involvement of reactive oxygen species (ROS) potentially of extra-mitochondrial origin in TPEN-induced LD death. Pentavalent antimonials remained the prime source of treatment against leishmaniasis for several decades; however, antimony-resistant *Leishmania* is now common source of the disease. We also reveal that Zn-depletion can promote apoptosis-like death in antimony-resistant parasites. In summary, we present a new finding about the role of zinc in the survival of drug sensitive and antimony-resistant LD.

## Introduction

Zinc as a cofactor of several hundred enzymes is integral for a wide variety of cellular processes that control numerous biological functions^1, 2^. Its bioavailability is important for cell proliferation, differentiation, regulation of apoptosis and other various cellular homeostasis mechanisms^3^. Zinc is an essential trace element in almost all kingdoms of life including humans^2^, animals^4^, plants^5^ and microorganisms^6^. It plays a structural role in zinc fingers, twists and clusters to regulate gene expression^7^. Zinc can be stored in metallothionein both in microorganisms and in the liver of animals^6, 8^, while inadequate zinc intake is also harmful to the organisms^6, 9^. The importance of zinc in biology is further underscored because it is the only metal that appears in all classes of enzymes^5^. As a redox-inactive metal it contributes to maintain cellular redox balance by various mechanisms^10^. Zinc is also well documented to be involved in apoptosis in mammalian cancer cells^3^ suggesting this micronutrient has various roles in maintaining crucial cellular mechanisms.

Leishmaniasis is a vector-borne disease that is transmitted by phlebotomine sand flies and caused by more than 20 species of obligate intracellular protozoa of the genus *Leishmania* ^11^. *Leishmania donovani* (LD) infection causes visceral leishmaniasis or kala-azar, which is associated with high mortality with an estimated 50,000 deaths each year^12^. The digenetic protozoan LD exists in two different forms in its life-cycle. One form is infective, flagellated promastigote, living in the gut of the sandfly and the other form is non-flagellated amastigotes residing in the mammalian host. The promastigote form is transmitted through sandfly bite and is taken up by macrophages in mammalian hosts. Inside the macrophage the promastigote is transformed into an aflagellated replicative amastigote. VL is widely prevalent in many tropical and subtropical regions of the world including the eastern part of India^13^. It is fatal if not treated properly. Moreover, drug resistance is a major problem in treating VL^14^. Current front line antileishmanial therapies are limited by their high costs, limited availability and/or toxicity and the widespread resistance in endemic areas^12^. Therefore, it is of utmost importance to look for effective new drugs for the treatment of leishmaniasis. Depriving essential micronutrients to parasites may be an effective way to control VL. Although zinc is a well established micronutrient for most of the organisms; however, the effect of its depletion on LD or any other *Leismania* species has not been reported so far.

Interestingly, zinc plays an integral part of virulence of *Leishmania* parasite. The promastigote form contains high gp63 protease that plays significant role in LD virulence. Zinc is an intergral part of gp63 as a cofactor^15^. Recently, the presence of Zn-transporter in *Leishmania infantum* has been reported underscoring the importance of zinc in crucial cellular functions of this parasite^16^. However, its role in growth and survival of any *Leishmania* species has not been reported so far. Here we report that Zn-depletion by specific chelator N,N,N′,N′-tetrakis(2-pyridinylmethyl)-1,2-ethylenediamine (TPEN) affects LD survival and growth by promoting cell death resembling apoptosis by a reactive oxygen species (ROS) dependent mechanism. We also reveal that antimony- resistant LD parasites are similarly affected by TPEN treatment. Our findings thus suggest an important role of zinc in survival of both drug-sensitive and antimony-resistant LD.

## Results

### Zinc chelation affects viability of LD promastigotes

**Figure 1:**
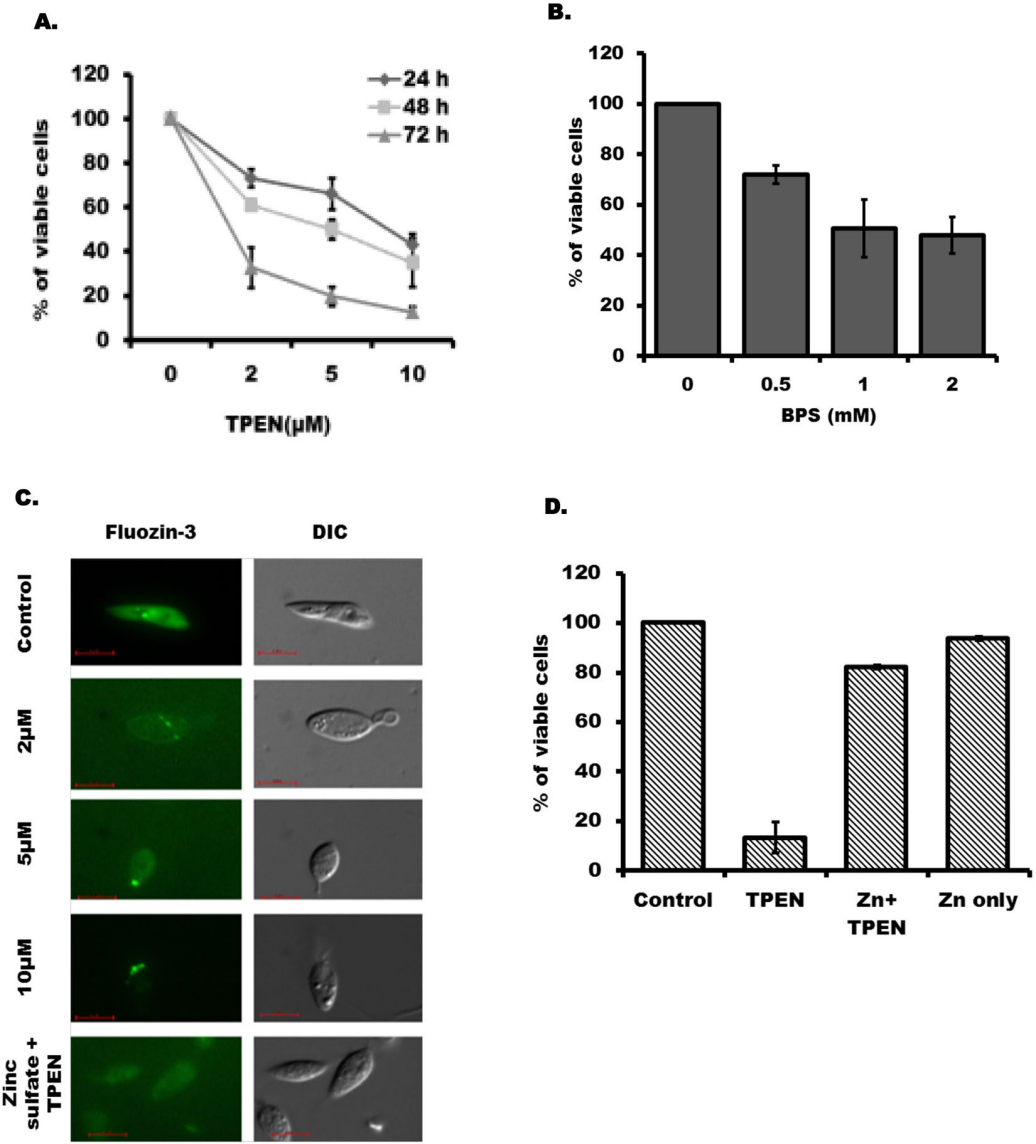
TPEN treatment affects viability of LD promastigotes: (**A**) Cell viability was verified by MTT assay after TPEN treatment (0–10 µM) at various time point (24–72 h). Results were expressed as percentage of untreated control of each time point performed independently from three experiments. (**B**) MTT assay was performed after 72 h of BPS treatment (0–2 mM). (**C**) Parasites were incubated with different concentrations of TPEN (0–10 µM) for 48 h. In one set, 10 µM zinc sulphate was added 1 h prior to TPEN (10 µM) treatment. After the treatment, parasites were stained with FluoZin-3^AM^ (5 µM) for 1 h. Left panel shows fluorescence-labelled *Leishmania* while right panel represents DIC image of the same parasite (Magnification × 100). (**D**) MTT assay was performed after 72 h of following treatments; Control, TPEN (10 µM), TPEN (10 µM) + Zinc Sulfate (10 µM) and Zinc Sulfate (10 µM). Zinc sulphate was added 1 h prior to TPEN treatment. Results are expressed as mean ± S.D. from three independent experiments performed in triplicate for A, B and D.

LD promastigotes were treated with increasing concentrations of zinc chelator TPEN (0–10 µM) for up to 3-days and viability of the parasites were verified by MTT assay. Result showed that time and concentration dependent increase in cytotoxicity of LD by TPEN treatment (Fig. 1A). We detected about 37%, 23% and 15% cells were viable after 72 h in response to 2 µM, 5 µM and 10 µM TPEN treatment respectively (Fig. 1A). The IC_50_ was detected as 4.78 µM for 48 h TPEN treatment. We also examined LD growth in a similar condition and about 85% decrease was detected after 72 h by 10 µM TPEN treatment compared to untreated parasites; while 2 µM and 5 µM TPEN reduced LD growth about 40% and 75% respectively. Since, iron is crucial for survival of *Leishmania* ^17, 18^ and TPEN is also known to chelate ferrous iron but with less affinity than zinc, so we further verified LD viability in presence of specific ferrous iron chelator BPS (4,7-Diphenyl-1,10-phenanthroline- disulfonic acid disodium salt trihydrate). Results showed that LD viability was affected by much higher concentration of BPS (Fig. 1B) compared to TPEN. Similarly, LD growth was affected only about 30% after 72 h treatment with BPS (1 mM). We used specific tetra anionic dye Fluozin-3^AM^ to determine whether TPEN treatment actually resulted in depletion of zinc. We detected decreased Fluozin-3^AM^ sensitivity with increasing concentration of TPEN treatment (Fig. 1C) suggesting TPEN actually could deplete available pool of intracellular zinc in LD. To further establish the role of zinc in LD survival, zinc sulphate (10 µM) was added along with TPEN (10 µM) and Fluozin-3^AM^ sensitivity as well as LD viability were examined. We detected almost complete reversal of Fluozin-3^AM^ sensitivity (Fig. 1C) and LD viability (Fig. 1D) by addition of zinc sulphate suggesting TPEN affected LD viability by chelation of available zinc. The result of MTT assay showed 82.2% of viability reversal from 13.4% by addition of zinc sulphate along with TPEN while only zinc sulphate treatment showed 93.8% viable cells (Fig. 1D).

### Zinc chelation promotes apoptosis-like death of LD promastigotes

**Figure 2:**
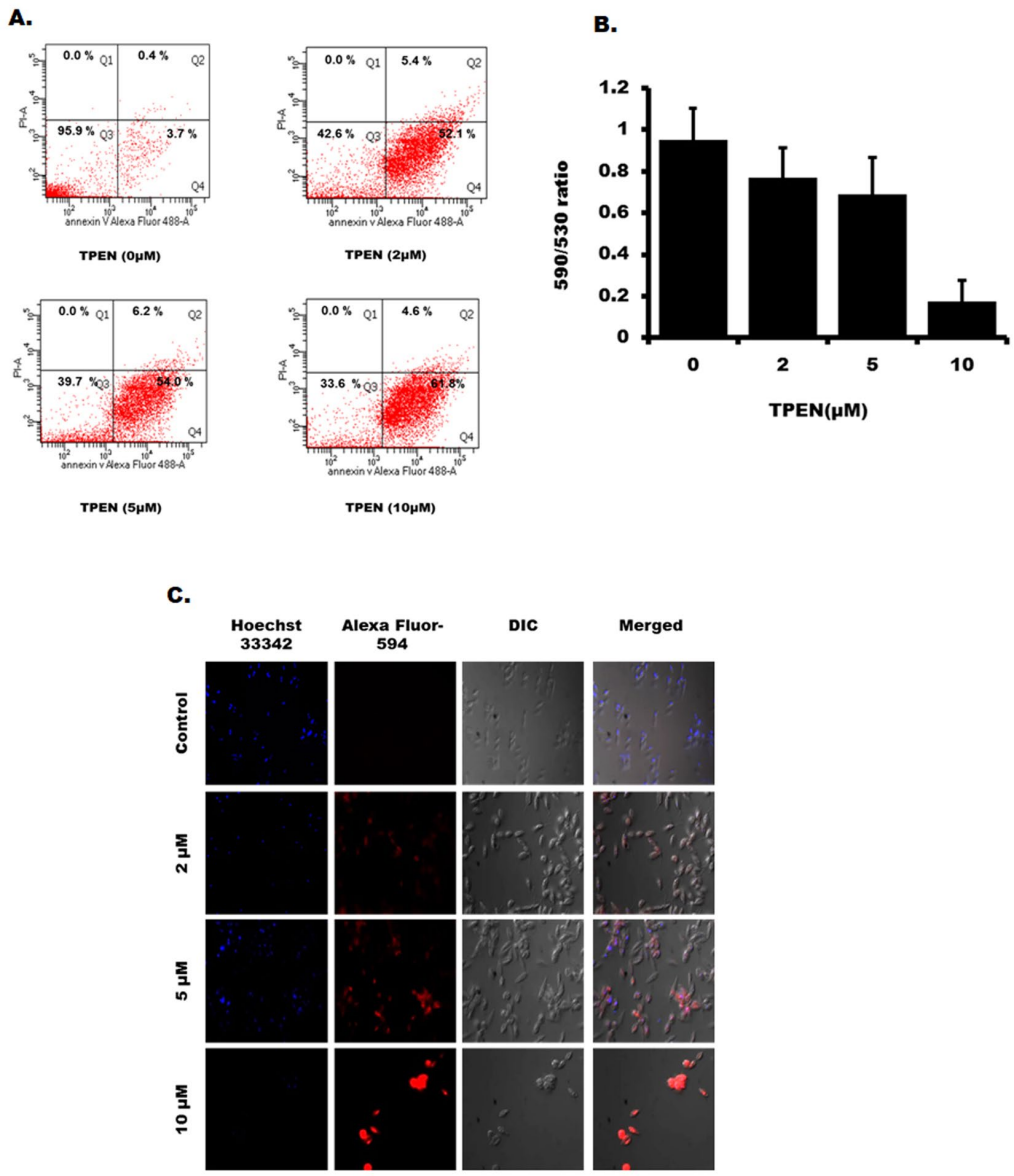
TPEN treatment promotes apoptosis-like death in LD. (**A**) Flow cytometric analysis for phosphatidylserine externalisation was detected by annexin V labelling in TPEN (0–10 µM) treated LD after 16 h. The left lower quadrant shows unstained parasite, right lower quadrant denotes annexin V positive cells, upper left quadrant shows only PI positive cells while upper right quadrant indicates both annexin V and PI positive cells. Dot plots are representative of one of three similar results. (**B**) Fluorometric analysis of mitochondrial membrane potential after TPEN treatment (0–10 µM, 24 h). Results represent as +/− SD from three independent experiments. (**C**) TUNEL staining was done in LD after TPEN treatment (0–10 µM, 72 h) for assessing DNA fragmentation. Microscopic images show red colour for TUNEL-positive LD (TdT staining) and blue colour for staining nucleus (Hoechst 33342) (magnification × 40).

Phosphatidylserine (PS) externalisation is one of the most remarkable features for apoptosis in almost all the cells. So we verified whether Zn-chelation induced LD death also featured PS externalization by using Alexaflour-488 conjugated-annexin v labelling. FACS analysis showed marked increase in the intensity of annexin v staining but not any significant staining of propidium iodide in TPEN-treated (16 h) parasites in comparison to control parasites (Fig. 2A). Mitochondrial membrane depolarisation is another important characteristic of apoptotic cell death. So, we further examined whether Zn-depletion could influence mitochondrial membrane depolarisation in LD by using JC-1 dye. Mitochondrial membrane depolarization was usually accompanied by a decrease in the fluorescence intensity ratio (590 to 530 nm). Results from TPEN-treated LD for 24 h showed decreased ratio of 590/530 in a concentration dependent manner (Fig. 2B) suggesting zinc depletion could affect mitochondrial membrane depolarization. These results suggest that zinc depletion can promote apoptosis-like events in LD within 24 h of TPEN treatment.

Activation of endonucleases and subsequent degradation of genomic DNA are hallmark and ultimate determinant of apoptosis that can be determined by TUNEL assay based on the incorporation of modified dUTPs by terminal deoxynucleotidyl transferase (TdT) enzyme at the 3′-OH ends of fragmented DNA^19^. Therefore, we performed TUNEL assay to confirm DNA fragmentation in TPEN-treated LD. We detected that TPEN even at 2 µM was able to cause DNA fragmentation in many cells and at 10 µM in almost all cells (Fig. 2C) further suggesting apoptosis-like death in Zn-depleted LD.

### Zn-depletion promotes caspase-like activity

**Figure 3:**
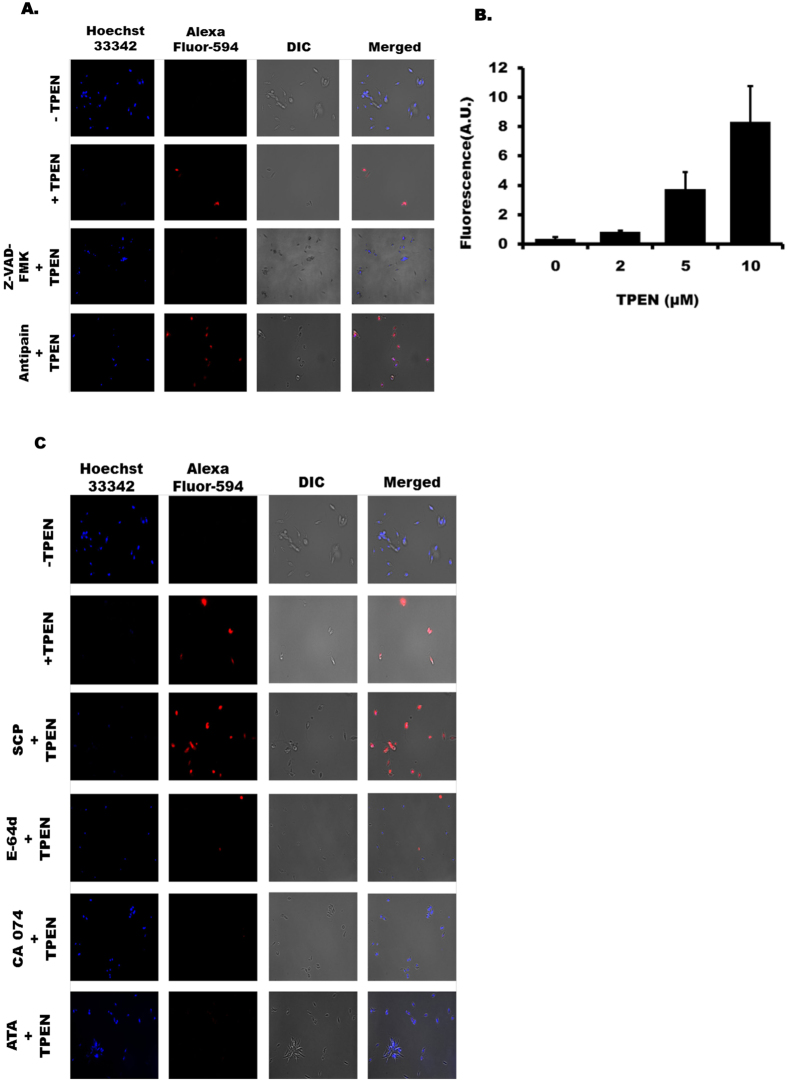
Zn-depletion promotes caspase-like activity and other proteases for LD death. (**A**) TUNEL assay was performed in LD after 48 h treatment with 10 µM TPEN, 10 µM TPEN + Z-VAD-FMK (10 µM, added 30 min prior to TPEN) and Antipain (50 nM, added 30 min prior to TPEN). Hoechst 33342 was used for staining nucleus (magnification × 60). Results represent one of the five independent experiments. (**B**) LD promastigotes were treated with TPEN (0–10 µM) for 48 h. Caspase 3/7 activity assay was performed in cell lysates as per company’s protocol. Results represent data from three independent experiments; error bars +/− SD. (**C**) TPEN induced DNA fragmentation was detected by TUNEL assay after 48 h. Parasites were treated with TPEN (0 or 10 µM). E-64d (10 µM), CA074 (10 µM), ATA (50 µM) and SCP (10 µM) were added 30 min prior to TPEN treatment. Hoechst 33342 was used for staining nucleus (magnification × 60). Results represent one of the four independent experiments.

Involvement of caspase-dependent and caspase-independent mechanisms of apoptosis has been well established in organisms^20–22^ and also advocated in *Leishmania* ^23–25^. Interestingly, the presence of caspases has not yet been confirmed in LD but the presence of caspase-like activity is reported earlier^26^. So, we examined the role of caspase-like activity in death of TPEN treated LD by prior incubation of parasites with general caspase inhibitor Z-VAD-FMK (10 µM). Results detected by TUNEL assay showed complete blocking of DNA fragmentation by Z-VAD-FMK (Fig. 3A) suggesting involvement of caspase-like activity in Zn-depletion induced cell death in LD. The presence of two metacaspases (1 and 2) is reported in LD but their precise roles are not yet well defined^27^. An earlier report revealed the role of metacaspase in disuccinyl betulin induced LD death^28^. So we investigated the role of metacaspase by pretreating LD with specific inhibitor antipain (50 nM). Antipain, which was reported to block disuccinyl betulin induced LD death^28^, was found ineffective in blocking TPEN induced DNA fragmentation suggesting metacaspases played little role in this process (Fig. 3A). Only Z-VAD-FMK or antipain did not show any influence on DNA fragmentation in LD (data not shown). Further we investigated the effect of TPEN on caspase-like activity in LD by performing caspase 3/7 activity assay. Results showed about 2.3, 10.7 and 23.7-fold increase in caspase3/7 activity after treatment with 2 µM, 5 µM and 10 µM TPEN (48 h) respectively in comparison to untreated parasite (Fig. 3B).

### Role of other proteases in TPEN-induced death in LD

It is now well-established that a number of proteases play crucial role in mediating apoptosis^29, 30^. Cysteine proteases like cathepsins also play important role in apoptotic cell death in mammalian cells^31, 32^. Therefore, we further explored whether any cysteine protease was involved in Zn-depletion induced death of LD. We performed TUNEL assay and found general cysteine protease inhibitor (E-64d) could rescue TPEN-induced DNA fragmentation in LD (Fig. 3C). To further detect involvement of any specific cysteine protease we used specific inhibitors of cathepsin B (CA074) and cathepsin L (SCP) and found that only cathepsin B inhibitor could rescue TPEN-induced DNA fragmentation but cathepsin L inhibitor showed no effect (Fig. 3C).

Mitochondrial nuclease Endonuclease G (Endo G) is reported to be involved in mitochondrial replication^33^ and DNA fragmentation in LD^24^. So, we further examined the role of Endo G in TPEN-induced LD death by using specific inhibitor aurintricarboxylic acid (ATA). ATA functions by inhibiting binding of the nuclease to DNA to block fragmentation^24^. TUNEL assay revealed that ATA could completely inhibit TPEN-induced DNA fragmentation in LD (Fig. 3C). To further determine the impact of cathepsin B and Endo G we verified growth rate of TPEN-treated LD in presence of their specific inhibitors (CA074 and ATA). Results showed affected LD growth by TPEN treatment was significantly reversed in presence of inhibitors of cathepsin B and Endo G (Supplemental Fig. 1). These experiments strongly suggest the role of cysteine protease cathepsin B and mitochondrial endonuclease (Endo G) in death process of LD due to Zn-depletion.

### Zn-depletion induces ROS generation to promote LD death

**Figure 4:**
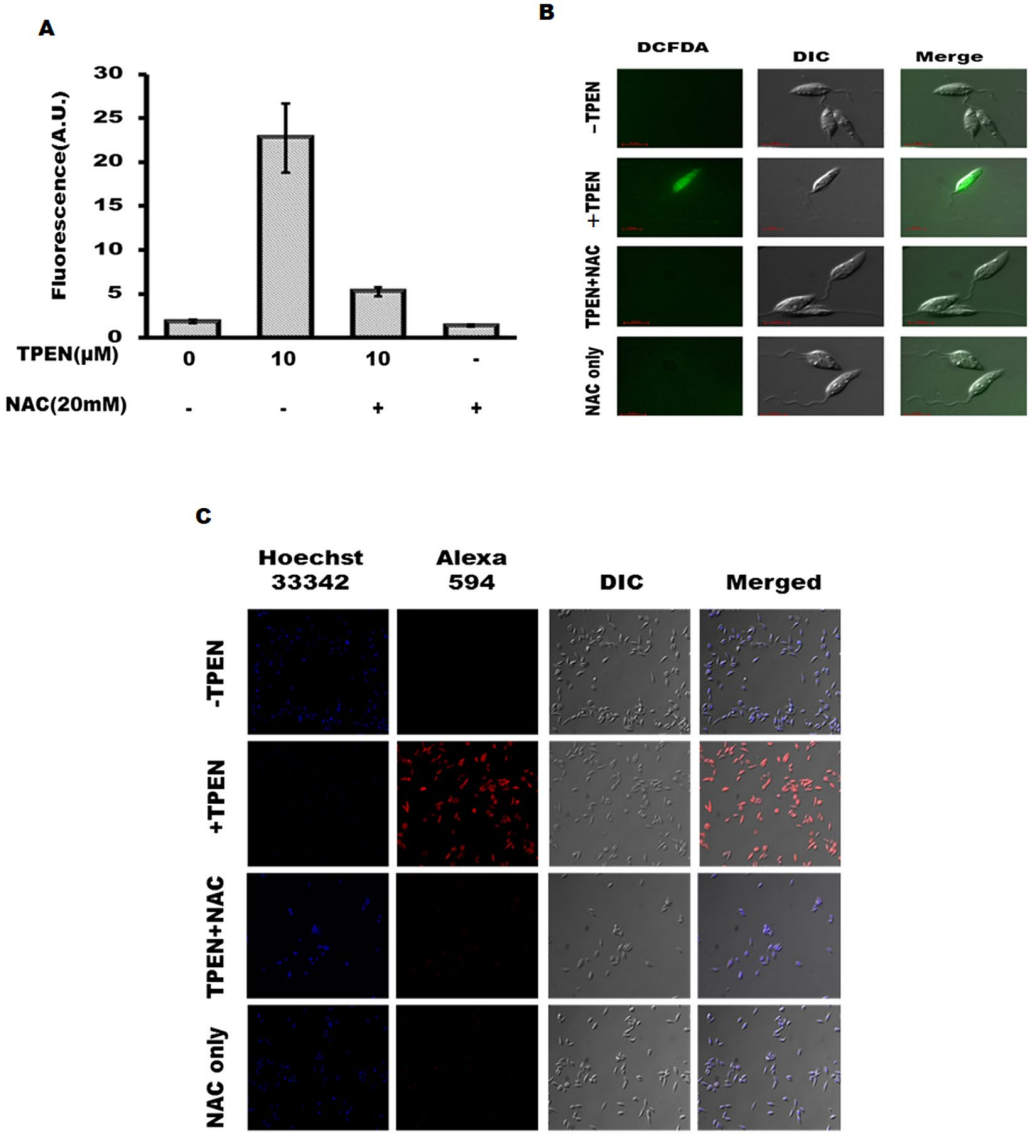
Zn-depletion induces ROS generation in LD. (**A**) DCF- fluorescence was measured in TPEN (0 and10 µM, 36 h) treated LD. NAC (20 mM) was added 30 min prior to addition of TPEN. Data represent mean ± S.D. from three independent experiments. (**B**) Similarly, ROS generation was detected by fluorescence microscopy (magnification × 100) at 24 h in presence of TPEN (0 and 10 µM) and NAC (20 mM). (**C**) TUNEL assay was performed in a similar condition described in (**A**) (magnification × 60). Data represent one of the three independent experiments.

ROS generation is suggested to be involved in promoting apoptosis in *Leishmania* parasites^34, 35^. Due to well documented role of zinc on cellular antioxidant capacity we considered that its depletion might increase ROS generation to promote apoptosis like events in LD. Therefore, we initially examined intracellular ROS level in TPEN treated LD. H_2_DCFDA was used to detect ROS level in TPEN treated LD promastigotes. A significant increase (~12 fold) in DCF-fluorescence was detected that was blocked by prior treatment of antioxidant NAC (N-acetyl cysteine) (Fig. 4A) suggesting increased ROS generation due to depletion of chelatable zinc pool in LD. We further verified ROS generation by fluorescence microscopy and found a similar result (Fig. 4B). To determine the role of ROS in Zn-depletion induced LD death process we performed TUNEL assay in presence of antioxidant NAC. Results revealed that NAC could protect TPEN-induced DNA fragmentation in LD (Fig. 4C).

### Zn-depletion does not induce mitochondrial ROS generation

**Figure 5:**
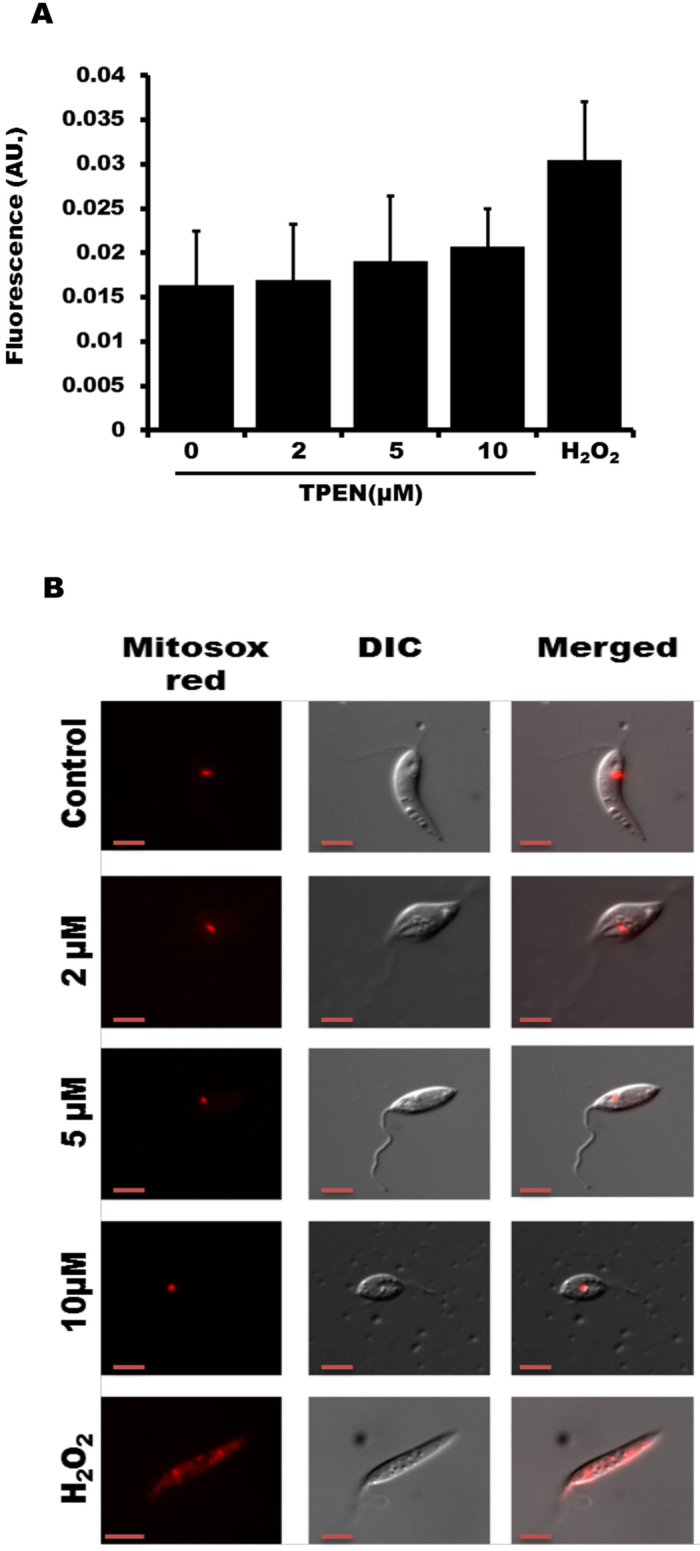
TPEN does not influence mitochondrial ROS generation. (**A**) LD parasites were treated with TPEN (0–10 µM) for 48 h. MitoSox (5 µM) was added 15 min prior to the end of incubation period. Fluorescence was measured at 590 nm to estimate ROS generation. Data represent +/− SD from three independent experiments. (**B**) Similarly, fluorescence microscopy (magnification × 100) was used to detect mitochondrial ROS generation using MitoSox. H_2_O_2_ (4 mM) treatment for 2 h was used as a positive control. Data represent one of the three independent experiments.

Mitochondrial ROS generation has been suggested as the primary source for apoptotic cell death in general and in *Leishmania* parasite^36, 37^. Since increase in DCF-fluorescence usually represents cytosolic ROS generation, we further estimated mitochondrial ROS using MitoSox as a probe. Results showed no significant change in mitochondrial ROS generation (Fig. 5A). H_2_O_2_ (4 mM, 2 h) was used as a positive control. A similar data was also obtained by using fluorescence microscopy (Fig. 5B). These results indicate that mitochondrial ROS generation has little role for promoting death in zinc depleted LD.

### Role of zinc chelation on viability of antimony resistant LD

**Figure 6:**
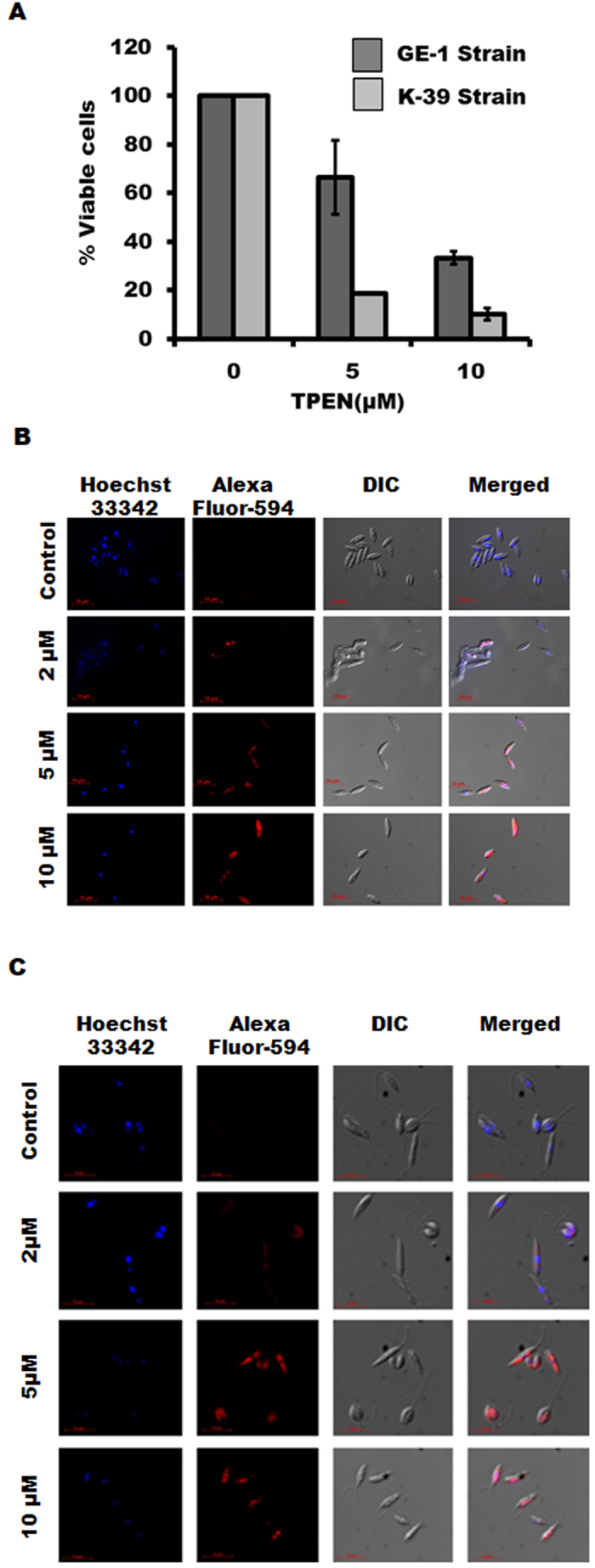
Effect of TPEN on drug resistance LD. (**A**) Cell viability of drug-resistant GE1 and K39 was detected after 48 h incubation with TPEN (0, 5 and 10 µM) by MTT assay. Data represent mean ± S.D. from three independent experiments performed in triplicate. TUNEL assay was performed in drug-resistant LD strains GE1 (**B**) and K39 (**C**) after TPEN treatment for 48 h (0–10 µM) (magnification × 60). Data represent one of the three independent experiments.

Our earlier experiments revealed that Zn-chelation could affect LD viability in wild type LD. So we further examined the effect of Zn-chelation on viability of antimony resistant parasites like GE1 [sodium antimony gluconate (SAG) resistant] and K39 (Potassium-Antimony-Tartarate resistant). MTT assay revealed the ability of TPEN on affecting viability of GE1 and K39 (Fig. 6A) substantially. IC_50_ for GE1 and K39 strain for 48 h TPEN treatment was detected as 7.18 µM and 3.82 µM respectively. The growth of these antimony resistant parasites was also decreased by about 90% after 3 days of TPEN (10 µM) treatment compared to untreated parasites. To verify whether Zn-depletion was also effective in promoting DNA fragmentation in these antimony resistant strains we performed TUNEL assay. Results showed TPEN treatment could promote DNA fragmentation in these drug resistant parasites (Fig. 6B and Fig. 6C). These results strongly suggest the effectiveness of Zn-chelation in promoting apoptosis-like death in antimony resistant strains of LD.

### TPEN treatment induces ROS generation to promote cell death in antimony resistant LD

**Figure 7:**
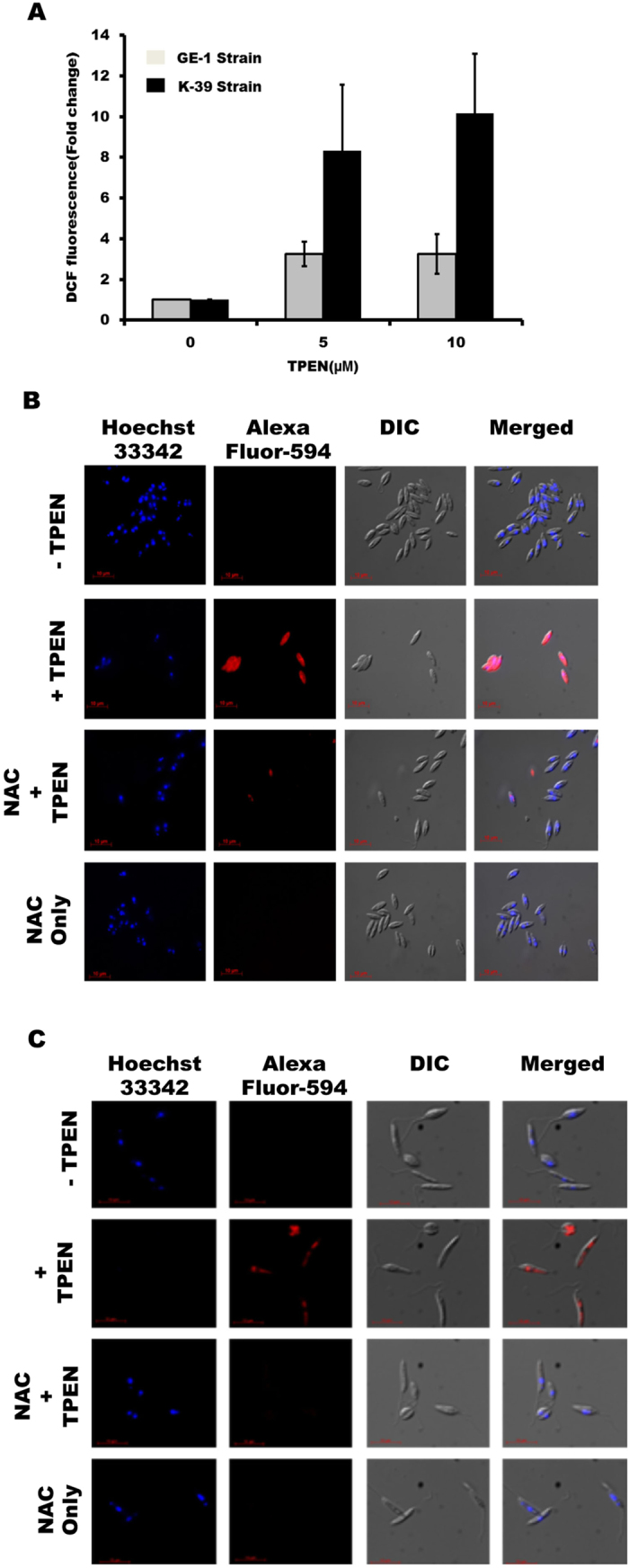
TPEN treatment induces ROS generation to promote apoptosis-like death in antimony-resistant LD. (**A**) ROS generation was estimated using H_2_DCFDA in GE1 and K39 LD parasites after 48 h of TPEN treatment (0, 5 and 10 µM). Data represent mean ± S.D. from three independent experiments. TUNEL assay was performed after TPEN treatment for 24 h (0 and 10 µM) in presence and absence of NAC (20 mM) in GE1 (**B**) and K39 (**C**) strains. Results are representative of one of the three independent experiments (magnification × 60).

Further we examined the effect of Zn-chelation on ROS generation in antimony-resistant GE1 and K39 LD strains. We detected substantial increase in DCF fluorescence in both the antimony-resistant strains of LD after TPEN treatment (Fig. 7A). We further observed that pre-treatment of antioxidant NAC blocked TPEN-induced DNA fragmentation by TUNEL assay (Fig. 7B and C). These results suggest Zn-depletion induced ROS generation play critical role in promoting death in antimony-resistant LD strains.

## Discussion

All microbial pathogens require transition metals for their growth and survival as these metals participate in many structural, catalytic and signalling functions. Zinc is one of these essential elements required for growth and virulence of pathogens^6^. The role of zinc in regard to growth and survival of *Leishmania* parasites has not been addressed despite being a significant component of the virulence armoury of these parasites particularly in its promastigote stage^15^. The current work is thus the first to report the crucial role of zinc in survival of LD. We demonstrate a novel finding that depletion of zinc could promote death of LD. Interestingly; we also found that two antimony-resistant LD strains were also susceptible to depletion of zinc like drug-sensitive parasites.

TPEN is widely used as cell permeable metal ion chelator mainly to limit intra- and extracellular concentrations of several transition metals; however, it is more effective in chelating zinc than ferrous iron^38, 39^. This study provides several evidences to show that TPEN actually chelated zinc to promote apoptosis-like death in LD. First, we found TPEN treatment affected LD viability and growth even in the presence of 10% serum that contained high amount of transferrin-bound iron, haemoglobin and other non-transferrin bound iron. Second, we detected specific ferrous iron chelator BPS could affect LD viability (Fig. 1B) but effectively in milliMolar concentration while TPEN was effective even at low (10 µM or less) concentration (Fig. 1A). Third, we found simultaneous addition of zinc salt along with TPEN could reverse the LD survival (Fig. 1D). Finally, we used an intracellular zinc sensor (Fluozin-3^AM^) to detect depletion of this transition metal in TPEN-treated LD (Fig. 1C). These experiments provided strong evidence that TPEN-induced death in LD was actually due to depletion of chelatable pool of zinc.

Apoptosis is one of the major pathways of cell death in organisms and also proposed to be part of the leishmanial death mechanism^37, 40^. However, existence of classical programmed cell death mechanism in protozoa including *Leishmania* is controversial as presence of many of the dedicated molecular pathways for apoptosis have not been identified yet^41^. We provided several evidences like phosphatidylserine externalisation, mitochondrial membrane depolarization, DNA fragmentation and involvement of Endo G in zinc depletion-induced death of LD (Figs 2 and 3) those recapitulated apoptosis-like cell death. Involvement of caspase-dependent and caspase-independent mechanisms for apoptosis has been well proposed in *Leishmania spp* ^42–44^. By using specific inhibitor we detected involvement of caspase or caspase-like activity in TPEN-induced LD cell death (Fig. 3A). We also detected a substantial increase in caspase3/7 activity by zinc depletion (Fig. 3B). Interestingly, the presence of caspase in *Leishmania* parasites has not been confirmed; however, evidence of caspase-like activity in this parasite is reported^45^. Thus, our observation is probably attributed to the presence of caspase-like activity in LD, the precise molecular identification of which is still not substantiated.

Apoptotic cell death is the result of increased activity of several other proteases in different organelles acting simultaneously and/or tandem^46^. Involvement of Endo G in leishmanial cell death is reported earlier^24^. There is also evidence of release of cathepsin B-like proteases from the lysosome that contribute to death of *Leishmania spp* ^47^. We found evidence of involvement of lysosomal protease cathepsin B and mitochondrial protease Endo G during zinc depletion induced death of LD. However, the precise mechanism by which zinc depletion induces these different proteases in different organelles is not known so far and needs further study.

ROS generation is a well established cause of apoptosis in general^48^ and also suggested in the *Leishmania* parasites^26, 49^. Given the suggested role of zinc as an antioxidant we assumed that zinc depletion might increase ROS generation in promoting death of LD. A strong increase in DCF-sensitive fluorescence was obtained due to TPEN treatment that was reversed by antioxidant NAC confirming that Zn-depletion could induce ROS generation in drug-sensitive and antimony-resistant LD (Fig. 4A,B; Fig. 7A). Further, we detected reversal of LD death by NAC (Fig. 4C; Fig. 7B,C) suggesting that depletion of zinc might affect antioxidant capacity to initiate death of LD. We limited our study with NAC up to 36 h because we detected toxicity after this period with only NAC treatment; the reason of which is not clear so far. However, there are previous reports on NAC toxicity in different cell types^50^ supporting our observation. Mitochondrial ROS generation is suggested as key event in promoting apoptosis-like death in LD by a number of agents including H_2_O_2_^26, 36^. We employed MitoSox, a sensor of mitochondrial ROS generation, and detected no significant alteration of MitoSox sensitivity suggesting ROS generation was from other cellular source than mitochondria due to zinc depletion (Fig. 5). Usually, increase in ROS-sensitive DCF-fluorescence is a measure of cytosolic ROS because the entry of DCF is mainly limited to cytosol^51, 52^. It needs further study to understand the precise mechanism by which Zn-depletion promotes ROS generation in LD.

One of the significant findings in this study is the ability of TPEN to induce ROS generation and apoptosis-like death in antimony-resistant LD. Most of the treatments of leishmaniasis so far have been centred on pentavalent antimonials^53^. Pentavalent antimonials like sodium antimony gluconate (SAG) are the standard first-line choice of drug against the disease^54^. We used two different strains of drug resistant LD; GE1 and K39 in the current study. GE1 strain is laboratory generated SAG-resistant strain^54^ while K39 is a clinical isolate of antimony resistant parasite^53, 54^. Earlier evidences suggested that most of the antileishmanial drugs functioned by promoting apoptotic death of the parasite, while drug resistant parasites became refractory to apoptosis^35^. In contrast, our findings suggest a very important fact that Zn-depletion could promote apoptosis-like death in antimony-resistant LD strains generated either in laboratory (GE1) or isolated clinically (K39). These results thus may open up possibility of zinc depletion as a strategy to develop antileishmanials towards drug-sensitive and antimony-resistant LD.

## Materials and Methods

Reagents were obtained from Sigma Chemical Company unless stated otherwise. Supplies related to tissue culture experiments were obtained from Corning, NY, USA. The MitoSox ^TM^ Red mitochondrial superoxide indicator, JC-1 mitochondrial potential sensor and Click-iT® Tunel Alexa Fluor® Imaging Assay from Molecular probes while Apo-One® Homogenous Caspase-3/7Assay kit was procured from Promega.

### Parasite culture

*Leishmania donovani* (MHOM\IN\1983\AG83) promastigotes were maintained in M199 medium supplemented with 10% FBS, 100 units/ml penicillin, 100 µg/ml streptomycin at 22 °C in BOD incubator as described earlier^17, 55^. Subculturing was done on every fourth or fifth day when the promastigotes reached the stationary phase of growth. Drug resistant parasites like K-39 (a clinical isolate resistant to PAT (SbIII)) and GE1 (a laboratory generated strain resistant to sodium antimony gluconate (SAG), the pentavalent antimony) were cultured as described earlier^54^.

### Study on LD growth

For growth study of LD, 1 × 10^6^ cells/ml were seeded into the 25 mL flask with fresh M199 with 10% FBS and 1% PS. Parasites were treated with different concentrations of TPEN and counted at every 24 h using neubauer chamber under a light microscope at 40X magnification. LD was counted from all four 16-big squares and an average was considered for further calculations. The formula used was; No. of cells = Average counting by neubauer chamber × dilution factor × 10^4^ cells/mL.

### Cytotoxicity assay

Viability of parasites was estimated using MTT (3-(4,5-dimethylthiazol-2-yl)-2,5-diphenyltetrazolium bromide) as described earlier^56^. Briefly, exponentially growing promastigotes (1 × 10^5^ cells/mL) in M199 media (without phenol red) with 10% FBS were treated with TPEN for different time points. After treatments cells were washed with ice-cold 1x PBS and incubated in fresh M199 media with 10% FBS and 400 µg/ml MTT. After 3 h, 100 µL DMSO was added to solubilise the formazan crystals. The absorbance was measured on a microplate reader (Sunrise, Tecan) at 492 nm. The percentage of viability was calculated from O.D. The blank O.D. was subtracted from all the samples. The viability of the cells was calculated using the following formula - viability of cells = (Absorbance of treated cells − Absorbance of Blank)/(Absorbance of control cells − Absorbance of Blank) × 100.

### Reactive oxygen species (ROS) generation

To investigate the level of endogenous ROS production, we used the peroxide-sensitive fluorescent probe H_2_DCFDA (Sigma-Aldrich). Briefly, parasites were washed with 1x PBS after treatment with TPEN and incubated with 50 µM probe for 30 min in dark at 22 °C. In the presence of endogenous superoxide, non-fluorescent membrane permeable H_2_DCFDA was converted into impermeable fluorogenic 2′,7′-dichlorofluorescein, which was detected fluorimetrically as described earlier^57^. Results indicate primarily the cytosolic ROS level induced by TPEN treatment. Similarly, ROS production was also verified by fluorescence microscopy using the probe H_2_DCFDA^58^.

### Mitochondrial ROS generation

For detection of mitochondrial ROS generation MitoSox ^TM^ Red mitochondrial superoxide indicator (Molecular Probe) was used as reported earlier^45^. It is live-cell-permeable, rapidly and selectively targeted to mitochondria. Once in the mitochondria, MitoSox^TM^ Red reagent is oxidized by superoxide and show red fluorescence. LD (1 × 10^6^ cells/mL) were treated with TPEN for 48 h at 22 °C. After treatment, cells were pelleted and washed with 1x PBS and further incubated with MitoSox Red reagent (5 µM) for 10 min. After incubation, cells were again washed with 1x PBS and transferred into 96 well plates. The fluorescence was measured in a fluorometer at ex/em of 543/590 nm. For microscopic detection, live leishmania cells were directly taken on the slide under cover-slip after washing with 1x PBS and examined under fluorescence microscope (Carl Zeiss-Axio-Vision-4.8).

### Caspase 3/7 activity assay

Caspase-like activity in LD was assayed with the help of a kit Apo-One® Homogenous Caspase-3/7Assay (Promega). The assay was performed according to manufacturer’s guidelines. Briefly, parasites after treatment were pelleted down and washed with 1x PBS. The pellet was resuspended in 1x PBS (50 µL) and mixed with caspase buffer in 1:1 ratio. The sample was then incubated for at least 12 h in dark at room temperature to get optimal fluorescence at an excitation wavelength range of 485 ± 20 nm and an emission wavelength range of 530 ± 25 nm.

### Estimation of mitochondrial membrane potential depolarisation

Mitochondrial membrane potential depolarisation was determined using mitochondria-specific probe JC-1 that could specifically accumulate within mitochondria according to membrane potential and provide fluorescence as per membrane potential^59^. To determine the mitochondrial membrane potential, parasites were treated with TPEN (0–10 µM), followed by washing with 1x PBS re-suspended in M199. Parasites were then incubated with JC-1 dye (10 µg/mL) for 20 min in dark. Then fluorescence was subsequently monitored in a fluorimeter (FLUOROSKAN ASCENT FL, Thermoscientific) at dual wavelengths as described earlier^59^. The result is expressed as the ratio of reading at 590 nm to the reading at 530 nm.

### TUNEL assay

We used Click-iT® Tunel Alexa Fluor® Imaging Assay kit (cat no. C10246) to detect DNA fragmentation. Experiments were performed as per company’s protocol. Briefly, parasites after treatment with TPEN were fixed with 3.7% formaldehyde for 15 min and then permeabilized with 0.25% Triton X-100 for 20 min. Equilibrium was created by adding 100 µL of TdT reaction buffer (Component A) for 10 min at room temperature. After removing TdT reaction buffer, 100 µL of TdT reaction cocktail was added for 1 h at 37 °C. Coverslips were washed for 3% BSA for 2 min. After that Click-iT reaction cocktail was added for 15 min. All samples were counterstained for nuclei with Hoechst 33342 and analyzed under a fluorescence microscope.

### Annexin-V binding Assay

Phosphatidylserine externalization was assessed using a kit from Molecular Probes (Invitrogen) as per company’s protocol. Briefly, parasites were harvested after treatment and washed with 1x PBS and resuspended in the 1x annexin V binding buffer according to cell density (1 × 10^6^ cells/mL). Alexa Fluor® 488 annexin v (component A) (5 µL) and 1 µL Propidium Iodide (100 µg/mL) were added to the sample. Samples were further incubated for 15 min at room temperature. After the incubation, 400 µL of 1x annexin V binding buffer was added to the cells and kept on ice until analyzed. Samples were analyzed by flow cytometry (within 1 h).

### Detection of intracellular zinc

Intracellular zinc level was detected with the help of sensor Fluozin™-3 (Molecular Probes) according to the protocol of the company. Fluozin™-3 is suitable for detection of Zn^2+^ concentrations. The cell-permeable AM-ester is useful for detecting low intracellular Zn^2+^ levels and small concentration changes^60^. Briefly, 1 × 10^6^ cells/mL were treated with zinc chelator TPEN for 48 h. Then cells were washed with1x PBS and resuspended in serum-free M199 medium (without phenol red). FluoZin™-3 (5 µM) was added to cells for 30 min at 22 °C in dark. Then cells were washed with only M199 (without phenol red) and further incubated for 45 min in dark at 22 °C to allow complete de-esterification of intracellular AM ester. After that cells were taken on glass slides and analysed under fluorescence microscope.

### Statistical analysis

All data are expressed as mean ± standard deviation (S.D.) and are represented as data of at least three different sets of experiments. Mean and standard deviation values were calculated with the help of Microsoft Excel.

## Electronic supplementary material

Zinc depletion promotes apoptosis-like death in drug-sensitive and antimony-resistance Leishmania donovani
