# Supplementary material for: Zinc depletion promotes apoptosis-like death in drug-sensitive and antimony-resistance Leishmania donovani
Source: Sci Rep. 2017 Sep 5;7:10488. doi: 10.1038/s41598-017-10041-6 (PMC5585245; doi:10.1038/s41598-017-10041-6)
Supplement: Supplementary file 1 — Zinc depletion promotes apoptosis-like death in drug-sensitive and antimony-resistance Leishmania donovani [file 41598_2017_10041_MOESM1_ESM.pdf]

Supplementary Information

Title: Zinc depletion promotes apoptosis-like death in drug-sensitive and antimony-resistance *Leishmania donovani*

Authors: Shalini Saini, Kavita Bharati, Chandima Shaha, Chinmay K. Mukhopadhyay

Supplementary Fig. S1 online

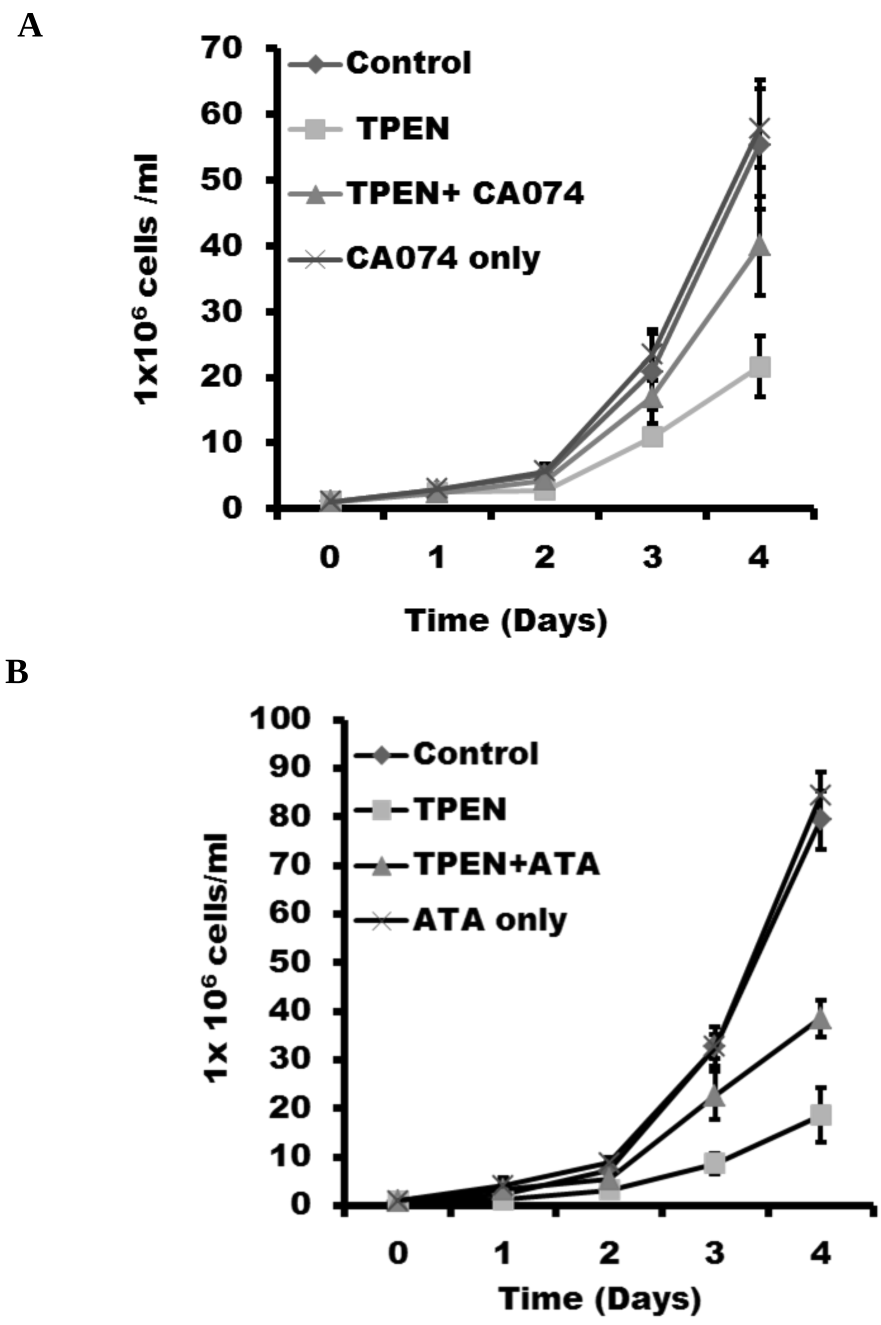

AG83 promastigotes ($1x10^6$) were treated with TPEN (0µM and 5µM) for 4 days in presence or absence of CA074 (10µM) (A) and ATA (50µM) (B). Numbers of parasites were counted at every 24 h using neaubar chamber. Data represented mean ± S.D. from three independent experiments.
